# Expression and regulation of HIF-1a in hypoxic pulmonary hypertension: Focus on pathological mechanism and Pharmacological Treatment

**DOI:** 10.7150/ijms.88216

**Published:** 2024-01-01

**Authors:** Jia-Jing Wan, Jian Yi, Fei-Ying Wang, Chao Zhang, Ai-Guo Dai

**Affiliations:** 1 School of Integrated Chinese and Western Medicine, Hunan University of Chinese Medicine, Changsha 410208, Hunan, People's Republic of China.; 2 Department of Respiratory Diseases, Medical School, Hunan University of Chinese Medicine, Changsha 410208, Hunan, People's Republic of China.; 3 Hunan Provincial Key Laboratory of Vascular Biology and Translational Medicine, Changsha 410208, Hunan, People's Republic of China.; 4 Department of Respiratory Medicine, First Affiliated Hospital, Hunan University of Chinese Medicine, Changsha 410021, Hunan, People's Republic of China.; 5 The First Affiliated Hospital of Hunan University of Chinese Medicine, Changsha 410021, Hunan, People's Republic of China.

**Keywords:** Hypoxia-inducible factor-1a, Hypoxia induced pulmonary hypertension, Pulmonary vascular remodeling, Right ventricular remodeling

## Abstract

Hypoxia inducible factor-1(HIF-1), a heterodimeric transcription factor, is composed of two subunits (HIF-1α and HIF-1β). It is considered as an important transcription factor for regulating oxygen changes in hypoxic environment, which can regulate the expression of various hypoxia-related target genes and play a role in acute and chronic hypoxia pulmonary vascular reactions. In this paper, the function and mechanism of HIF-1a expression and regulation in hypoxic pulmonary hypertension (HPH) were reviewed, and current candidate schemes for treating pulmonary hypertension by using HIF-1a as the target were introduced, so as to provide reference for studying the pathogenesis of HPH and screening effective treatment methods.

## 1. Introduction

The acute reduction of oxygen in the lung can lead to hypoxic pulmonary vasoconstriction (HPV). However, chronic hypoxic stimulation can cause to continuous contraction of pulmonary vessels, hypoxic pulmonary vascular remodeling (HPVR), and an increase of pulmonary circulatory resistance, resulting in the development of pulmonary hypertension (PAH). This change is largely irreversible^1^. The data of human subjects and animal models show that hypoxia-inducible factor-1 (HIF-1) plays a role in acute and chronic hypoxic pulmonary vascular reactions^2^. Combined with hypoxia response elements (HRE) in promoter region of target gene, the expression and regulation of HIF-1a enable organisms to cope with reduced partial pressure of oxygen in environment. However, prolonged activation of HIF-1a can give rise to changes in the structure of pulmonary blood vessels, thereby leading to the occurrence of PAH. PAH is characterized by an increase in pulmonary hypertension associated with pulmonary artery remodeling, accompanied by a reduction in the area of vascular lumen, and right heart hypertrophy; Eventually, the patient dies of right heart failure^3^. The apoptosis and proliferation imbalance of pulmonary artery smooth muscle cells (PASMCs) are considered to be the main link of hypoxic pulmonary hypertension (HPH), while HIF-1a plays an important role in the expression of various angiogenin and growth factors induced by hypoxia^4^. Semenza et al. found an oxygen-dependent nuclear transcription factor HIF-1 during the hypoxia treatment of hepatocellular carcinoma Hep3B in 1992^5^. It is a DNA-binding protein that can regulate the transcription of erythropoietin (EPO) gene. Previous researches on HIF-1a mainly focus on tumors. At present, the correlation among hypoxia, HIF-1a and chronic respiratory diseases is one of the current research hotspots^6^. It is critical to identify new candidate schemes for treating HPH with HIF-1a as the target by understanding the function and mechanism of HIF-1a expression and regulation in HPH.

## 2. Structure and function of HIF-1a

HIF-1 including subunits HIF- 1α and HIF- 1β is a seterodimer transcription factor^7^, of which HIF- 1α is a functional one. As a regulatory and active subunit, it is only expressed in the nucleus under hypoxia^8^.O2 mostly regulates the activity of HIF-1 by this subunit. As aryl hydrocarbon receptor nuclear transporter (ARNT), HIF-1β is also a subunit of aromatic hydrocarbon receptor compound, which can be expressed under normoxia and hypoxia, and interact with HIF- 1α to form a dimer. However, which aspects HIF-1β plays its role in is not clear. Currently, it may be related to the active conformational transformation caused by the dimerization of HIF-1 and its stability^9^.

**Figure 1:**
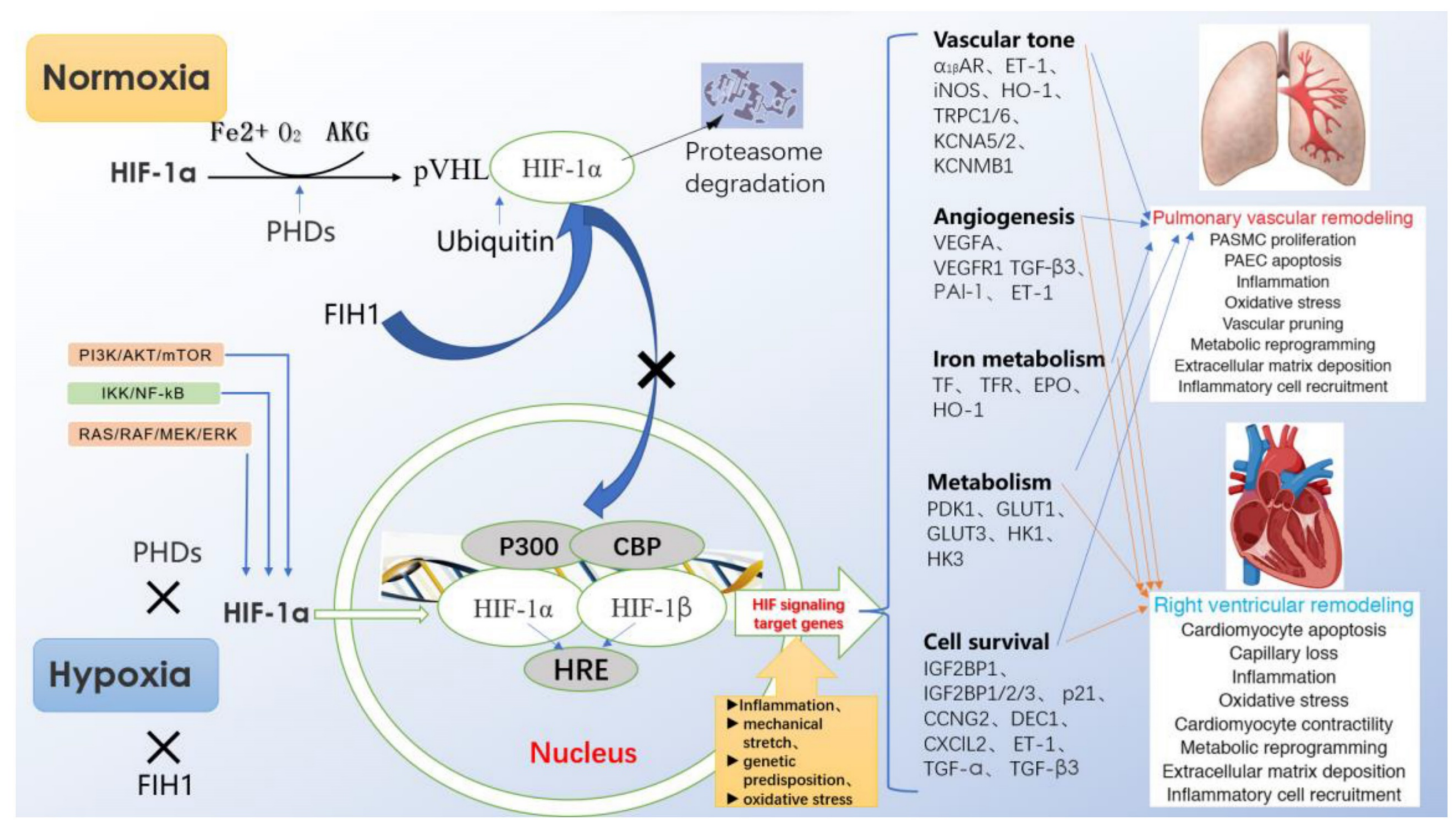
Regulation of HIF-1α expression, transcriptional activation pathways, and downstream target gene expression.

Under normoxia, HIF-1α hydroxylated by proline hydroxylase-domain protein (PHD) is quickly recognized by ubiquitin-E3 ligase compound, so that HIF-1α is rapidly degraded by ubiquitin proteasome^10^. However, under hypoxia, the effect of PHD is inhibited, so that HIF-1α can easily enter the nucleus to form a stable heterodimer structure with HIF-1β, and bind to the HRE of the gene. Among them, CBP/P300 protein is used as a bridge to connect RNA polymerase to induce the expression of the target gene. Activated HIF-1a can induce the expression of endothelin (ET), erythropoietin (EPO), vascular endothelial growth factor (VEGF) and inducible nitric oxide symthase (iNOS), and participate in the regulation of cell proliferation, angiogenesis, cell metabolism and inflammatory reaction (Fig. 1).

## 3. HIF-1a and HPH

HPH is a complex heart and lung disease caused by long-term hypoxia, which often occurs in chronic obstructive pulmonary diseases, interstitial pulmonary diseases and sleep apnea. Main clinical manifestations are exertional dyspnea, asthenia syncope, and sometimes angina pectoris, and clinicopathologic features are right heart overload, right ventricular hypertrophy, etc. Severe patients may have right heart failure, even die. Pathological mechanism of HPH is complex, which is mainly pulmonary vascular continuous contraction and irreversible remodeling under hypoxia^11^.

Under hypoxic condition, the synthesis of vasodilator and vasoconstrictor substances in the body decreases and increases respectively, resulting in a vasoconstrictive response; HIF-1a binds to the specific site of ET gene, which promotes the synthesis and secretion of ET by PAEC. ET-1 is a highly effective vasoconstrictor factor, and the imbalance of ET and NO expressions under hypoxia exposure is closely related to the occurrence of HPH. The changes of intracellular concentration of K^+^ and Ca^2+^ play an important role in regulating the contraction of PASMC. ET binds to ET-A on vascular smooth muscle cells and causes the contraction of PASMC through calcium dependent regulation and membrane depolarization^12^. Meanwhile, under long-term hypoxia exposure, ET can inhibit the binding of HIF-1a to the specific site of inducible nitric oxide synthase (iNOS) target genes, and the expression of iNOS mRNA, which causes a decrease in NO released from endothelial cells, and an imbalance between ET and NO production^3^. In addition, under hypoxia, the production of mitochondrial ROS also increases. ROS acts as a signaling molecule to activate HIF-1α and voltage gated calcium channel, and inhibit the expression of voltage gated potassium channel, which leads to an increase in intracellular concentration of Ca^2+^, and the contraction of smooth muscle cells^13^. Moreover, under continuous hypoxia, Rho kinase can be activated, and pulmonary vasoconstriction and HIF-1α expression can be enhanced^14^. However, under hypoxic stimulation, HIF-1a promotes an increase in the concentration of Ca^2+^ in PASMC. However, the specific mechanism of phenotypic changes remains to be explored.

The cellular structures and pathological processes involved in pulmonary vascular remodeling include the proliferation, migration, and dedifferentiation of pulmonary arterial smooth muscle cells (PASMCs); excessive proliferation and anti-apoptosis of pulmonary vascular endothelial cells (PAEC); proliferation and migration of fibroblasts; and macrophage aggregation. Further, the muscularization of non-muscular arteries, extracellular matrix deposition, and chronic inflammation are also observed, among other processes^15^, ^16^, ^17^, ^18^. In addition to pulmonary vascular remodeling, there is also obvious right ventricular remodeling (RVR). The following primarily introduces the impact of HIF-1α expression regulation on the above mechanisms.

### 3.1 HIF-1a and PASMCs

The proliferation and migration of PASMCs are closely related to pulmonary vascular remodeling. However, the regulation of HIF-1α under hypoxia is the key to affect angiogenesis and structural remodeling. Previous studies show that factors, such as vascular endothelial growth factor (VEGF), have a significant influence on the proliferation of vascular smooth muscle cells and the germination of new blood vessels^19^. It is found in the latest studies that a variety of cytokines, microRNA, non-coding RNA (ncRNA), glucose metabolism, oxidative stress, etc. can regulate the expression of HIF-1a^20^.

HIF-1α and heme oxygenase-1(HO-1) are important transcriptional regulatory factors in hypoxic cells and in maintaining cellular homeostasis. ZHANG et al.^21^ investigated the distribution of HIF-1α and HO-1 in the lungs of yaks. Immunohistochemistry and immunofluorescence results showed that HIF-1α and HO-1 are mainly concentrated in the middle layer of small pulmonary arteries and are significantly upregulated in hypoxic PASMCs. CD146 is significantly up-regulated in PASMCs, which is proportional to the severity of HPH. Destroying the interaction between CD146 and HIF-1α by gene ablation can weaken pulmonary vascular remodeling in chronic hypoxia model mice^22^. Another study indicates that HIF-1α mediates excessive proliferation, anti-apoptosis and calcification of PASMCs in pulmonary hypertension through the activation of Runt-related transcription factor 2 (RUNX2)^23^. Wang et al.^24^ found that the production of reactive oxygen species (ROS) increased and the expression of HIF- 1α was regulated under hypoxia, thus affecting the proliferation of PASMCs. Among them,2-methoxyestradiol can significantly improve the damage of mitochondria under hypoxia, and weaken the level of ROS in serum. It can also decrease HIF-1α in pulmonary tissue and vessels, inhibit pulmonary vascular remodeling and reduce pulmonary hypertension. Under hypoxia, the expression of KLF5 in PASMCs increases and both the expression of HIF-1α and the proliferation of PASMCs are promoted; silencing the expression of HIF-1α by small interfering RNA (siRNA) has no effect on the expression of KLF5, but weakens the proliferation ability of PASMCs^25^. The increased of SENP-1 expression can significantly up-regulate the HIF-1α expression and promote the proliferation of PASMCs under hypoxia^26^.

Various cytokines, microRNA and ncRNA participate in the regulation of HIF-1a expression under hypoxia. MiR-204 is primarily expressed in PASMCs, and its expression levels are downregulated in the chronic hypoxia and MCT-induced PH rat models^27^.Reduced miR-204 expression can activate HIF-1α, leading to PASMC proliferation and resistance to apoptosis^28^,while restoring miR-204 expression significantly reduces the severity of PH^29^. ET-1 and HIF-1α levels in serum of HPH patients are positively correlated with pulmonary artery systolic pressure (PASP)^30^. A study shows that HIF-1 regulates the expression of ET-1 by microRNA-543, and the reaction of pulmonary vessels to hypoxia^31^. Wang et al.^[32]^proved that mRNA levels of ET-1, HIF-1α and adrenomedullin (ADM) in neonatal rats during early hypoxia increased continuously, and mean pulmonary artery pressure and pulmonary vascular remodeling also rose. It is suggested that HIF-1α may regulate levels of ET-1 and ADM, thereby participating in the formation of neonatal HPH. Tang et al.^[33]^found that MicroRNA-143-5p regulated the function of PASMCs in HPH by targeting HIF-1α, and the over-expression of MicroRNA-143-5p significantly lowered the level of specific contraction marker protein and cell apoptosis of vascular smooth muscle, and improved the cell migration of PASMCs under hypoxia. Chen et al. observed^34^ that microRNA- 150 was down-regulated in hypoxic PASMCs and over-expressed HIF-1α weakened the inhibitory effect of miR-150 on the proliferation and migration of PASMCs. It can be concluded that miR-150 may inhibits the proliferation and migration of PASMCs by down-regulating HIF-1α. In addition, microRNA-195-5p secreted by apoptosis-resistant pulmonary microvascular endothelial cells (pecs/AR) has also been proved to promote the proliferation and migration of PASMCs in patients with PAH. Experiments have confirmed that MiRNA-195-5p, as a paracrine factor of the interaction between pecs/AR and PASMC, may play a role through HIF-1a/miR-195-5p/Smad7 pathway^35^. Under hypoxia, the down-regulation of lncRNA Rps41 expression in PASMCs leads to the increase of ILF3 and HIF-1α levels, and promotes the proliferation, migration and cell cycle process of PASMCs^36^.

Recent research reveals that hypoxia elevates a novel circular RNA, circ-myh8, which acts as a modular scaffold, recruiting histone acetyltransferase KAT7 to the promoter of the HIF-1α gene and subsequently inducing PASMC proliferation and cell cycle progression. The study posits that circ-myh8 and its associated pathways may serve as pivotal targets for the diagnosis and treatment of HPH^37^.

In HPH, PASMC transforms from normal oxidative phosphorylation of glucose to glycolytic reprogramming of glucose metabolism, which is extremely relevant to the abnormal activation of HIF-1α^38^. In the HPH animal model, it is found that the increase in the glycolysis and FDG uptake and expression level of glycolytic enzymes (GLUT1, PKM2, PFKFB3, HK1, etc.) in the lung and right ventricle is all connected to the increased expression of HIF-1α, indicating that HIF-1α is the key to promoting the glycolysis of PASMC under hypoxia^39^. Under hypoxia, the expression of glucose-6-phosphate dehydrogenase (G6PD) in PASMCs increases, which promotes expression of HIF-1a in PASMCs and lung tissues, and proliferation of PASMCs cells and remodeling of pulmonary vascular structure^40^, suggesting that the change in glucose metabolism may participate in the pathogenesis of HPH.

Xiao et al.^[41]^found that platelet-derived growth factor (PDGF) regulated HIF-1α expression through PI3K signal pathway, and promoted PASMCs proliferation. Ahmed et al.^[42]^revealed that oxidative stress might up-regulate transcription and translation of HIF-1α, which promoted PASMCs proliferation and participated in HPH formation. Moreover, Chen et al.^[43]^investigated that HIF-1α regulated mitochondrion division by directly up-regulating the expression of dynamic-related protein 1(Drp1), thus promoting the proliferation of PASMC and inhibiting its apoptosis under hypoxia. In lung sections or normal PASMCs, CoCl_2_ stabilizes HIF-1α, leading to DRP1-mediated mitochondrial fission^44^.These findings substantiate that the activation of HIF-1*α* mediates mitochondrial fission, thereby promoting PASMC proliferation^45^.

### 3.2 HIF-1a and PAECs

Endothelial cells, an indispensable barrier, can protect the vascular wall from damage caused by exogenous pathogens, etc.^46^ Recent studies show that endothelial dysfunction is an important sign of vascular remodeling^47^. As the earliest cell to perceive the change of oxygen content, PAEC is stimulated by hypoxia to stimulate its secretion function and regulate the proliferation process of itself and adjacent cells. In the pathogenesis of PH, PAECs may initially undergo apoptosis, but subsequently switch to an anti-apoptotic hyperproliferative state, which is related to the formation of plexiform lesions in the lungs of PAH patients and the pathological vascular remodeling in PH^48^,^49^.

Estradiol (17 β-Estradiol, E2) alleviates HPH through dependent effects of estrogen receptor (ER), including inhibiting the proliferation ofhypoxia induced endothelial cell. Andrea et al.^[50]^further confirmed that the protective effect of E2 in HPH was mediated by HIF-1α dependently increasing the expression of ERβ.In hypoxic PAEC, the stability of HIF-1α increases the ERβ of PAEC, while the down-regulation of HIF-1α decreases the abundance of ERβ;in addition, the down-regulation of ERβ also reduces the expression of proline hydroxylase domain 2(PHD2),a HIF inhibitor, while the activation of ERβ increases PHD2, and decreases HIF-1α and HIF-2α.It is indicated that the ERβ adjusts the shaft of PHD2/HIF-1α/HIF-2α under hypoxia. Wang Le et al.found that improving the expression of Hsp70 in PAEC of HPH newborn rats by adenovirus transfection promoted the degradation of HIF-1α, and down-regulated the expression of its downstream target genes ET-1 and iNOS, so as to reduce pulmonary artery pressure and alleviate pulmonary vascular remodeling.

In vivo, endothelial cells show different phenotypes according to local conditions. The interaction between endothelial cells and smooth muscle cells (SMCs) is the key mechanism of PH development^51^. Mesenchymal cells and SMCs are included in the reconstructed pulmonary vascular lumen, while cell markers of endothelial cells are not prominent. This phenomenon is called endothelial-to-mesenchymal transition (EndMT). Through EndMT, endothelial cells obtain the phenotype of mesenchymal cells and SMCs, but lose the phenotype of endothelial cells. Endothelial mesenchymal transforming cells can participate in vascular remodeling in PAH by directly transforming into SMCs with high proliferation and migration ability, and affecting the proliferation of vascular intima and media through paracrine^52^, ^53^. At the same time, EndMT can enhance the migration ability of PAEC and transform the slowly proliferating PAEC into a highly proliferating cell type, thereby resulting in the formation and development of occlusive endometrial lesions^54^. Studies have shown that^[55]^the co-localization of CD31 and its smooth muscle actin (α-SMA)of mesenchymal markers in the intimal layer α- of pulmonary arterioles in rats with chronic hypoxia is significantly down-regulated, and the expression of CD31 in cultured pulmonary microvascular endothelial cells (PMVECs) under hypoxia is significantly reduced; meanwhile, the expression of α-SMA and collagens Col1A1 and Col3A1 of other two mesenchymal markers is significantly increased; and inhibiting HIF-1α can effectively suppress the hypoxic induction of α-SMA, Col1A1 and transcription factor Twist1, and save the hypoxic inhibition of CD31 as well, indicating that in pulmonary artery remodeling, HIF-1α/Twist1 pathway significantly mediates the role of hypoxia induced EndMT for which HIF-1α is essential.

**Figure 2:**
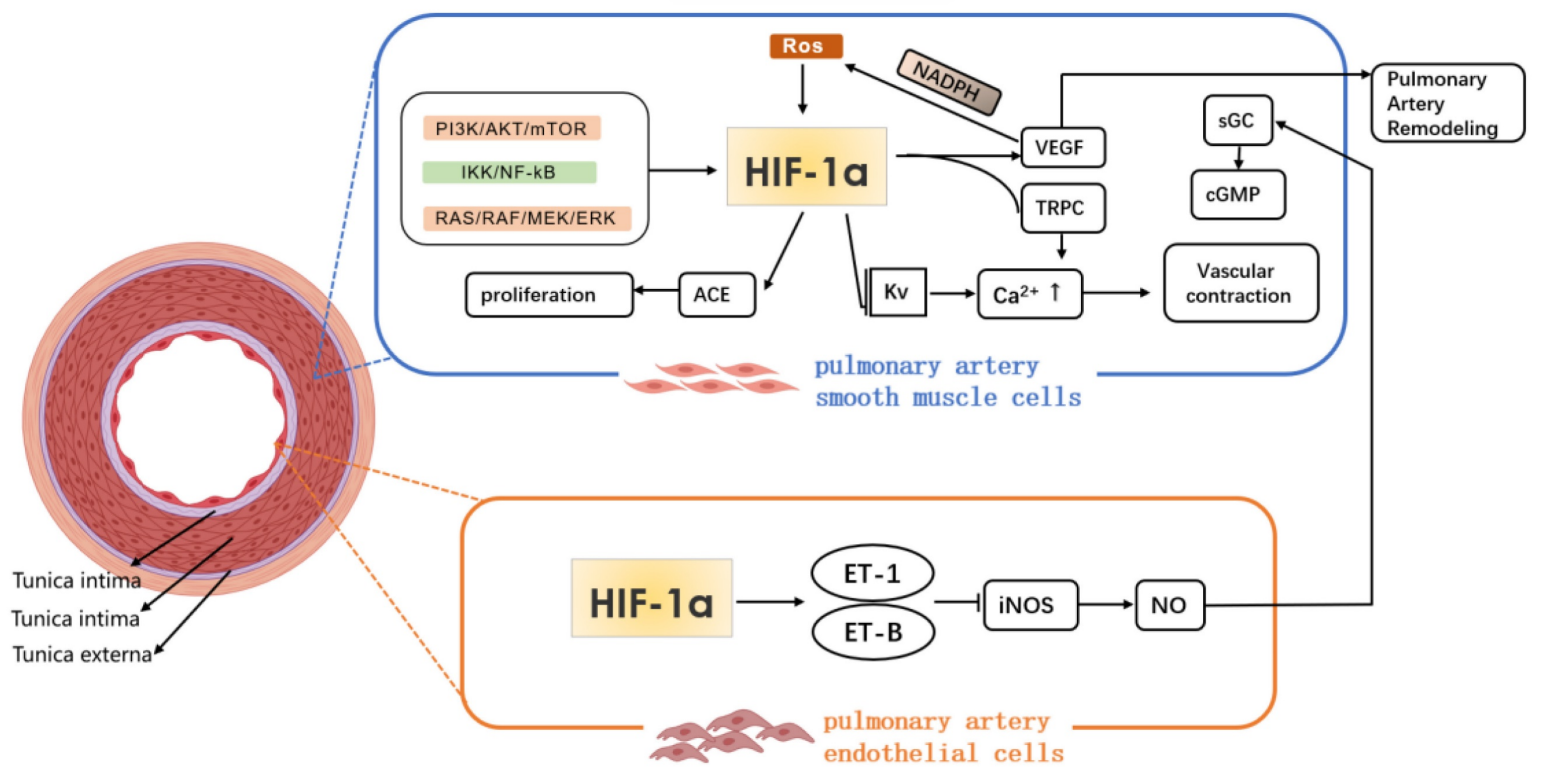
Mechanism of HIF-1α in pathological process of hypoxia pulmonary hypertension.

In recent years, it has been found that endothelial cell-specific molecule-1 (ESM-1) released by endothelial cells is a new regulatory factor related to vascular remodeling. ESM-1 is the downstream factor of VEGF. HIF-1α/VEGF can activate ESM-1, promote the adhesion between monocytes and endothelial cells, induce endothelial cell dysfunction and vascular remodeling, and may be involved in airway remodeling^56^. Moreover, IL-33, as a key inducing cytokine, is helpful for many pulmonary diseases. Liu et al.^[57]^found that the increase of IL-33 induced the proliferation, adhesion and angiogenesis of PAECs through the combination of its receptor ST2 and the activation HIF-1α/VEGF signaling pathway, and promoted vascular remodeling under hypoxia (Fig. 2).

### 3.3 HIF-1a and lung fibroblasts

Pulmonary vascular remodeling is mainly manifested by pulmonary vascular intimal hyperplasia, medial hypertrophy, adventitial fibrosis and inflammatory cell infiltration^58^. Studies have shown that the adventitia of pulmonary artery is the first structure to respond to vascular stress and injury with pathological changes^59^. Under the action of hypoxia, vasodilation and other factors, the pulmonary artery adventitia fibroblasts (PAAFs) are first activated, which is mainly manifested by significantly enhancing the proliferation and migration ability of the cells, and transforming into myofibroblasts, producing more extracellular matrix(ECM),growth factors, chemokines and inflammatory cytokines, regulating the growth of vascular wall cells, and participating in the process of pulmonary vascular remodeling^60^. In a study of PAH rats and COPD patients, it is found that inhibition of pyruvate dehydrogenase kinase (PDK), which is highly expressed in right ventricular fibroblasts, can accelerate degradation of HIF-1α, inhibit right ventricular fibrosis and hypertrophy, and improve right ventricular functions^61^.

In terms of genes, HIF-1α can also express HRE on α-SMA gene by combining myofibroblasts, induce airway fibroblasts to differentiate into myofibroblasts, and participate in hypoxic pulmonary vascular remodeling induced by COPD^62^. A study shows^63^ that hypoxia induced PAAF is accompanied by a sharp decline in miR-29a-3p; after HIF-1α is knocked out, the increase in miR-29a-3p of adventitial cells of posterior vessels promotes the proliferation and migration of PAAF and the expression of α-SMA and extracellular matrix protein, significantly reduces pulmonary artery pressure, right ventricular hypertrophy index, and improves pulmonary vascular remodeling. It is indicated that HIF-1α plays an important role in regulating the proliferation of PAAF, but its mechanism is still unclear. In addition, by adopting RNA interference technique to inhibit HIF-1 specifically, it is found that the migration reaction of PAAF is related to HIF-1α^64^. Interestingly, a recent study has discovered that even under normoxic conditions, human PAAF demonstrates a reduction in HIF-1α hydroxylation and an augmentation in the expression of HIF target genes. The study suggests that merely inhibiting HIF is insufficient to reverse the “persistently activated” phenotype observed in both human and bovine PAAF^65^.

In summary, hypoxia-induced fibroblast differentiation into myofibroblasts, mediated by the HIF-1α signaling pathway, is a component of hypoxia-induced PAAF proliferation. HIF-1α can serve as a therapeutic target to reduce the formation of these activated fibroblasts, thus reducing the formation of new intima and vascular remodeling during the HPH process. The specific mechanism of this process still requires further investigation.

### 3.4 HIF-1a and pulmonary macrophages

The role of immune inflammatory reaction in PAH vascular remodeling has attracted more and more attention. Macrophages with high heterogeneity and plasticity derived from monocytes are an important member of the reaction, and important inflammatory cells that cause pulmonary vascular remodeling. Under various conditions, they differentiate into multiple subtypes, and mediate a variety of biological effects, that is, the macrophage polarization^66^.

**Figure 3:**
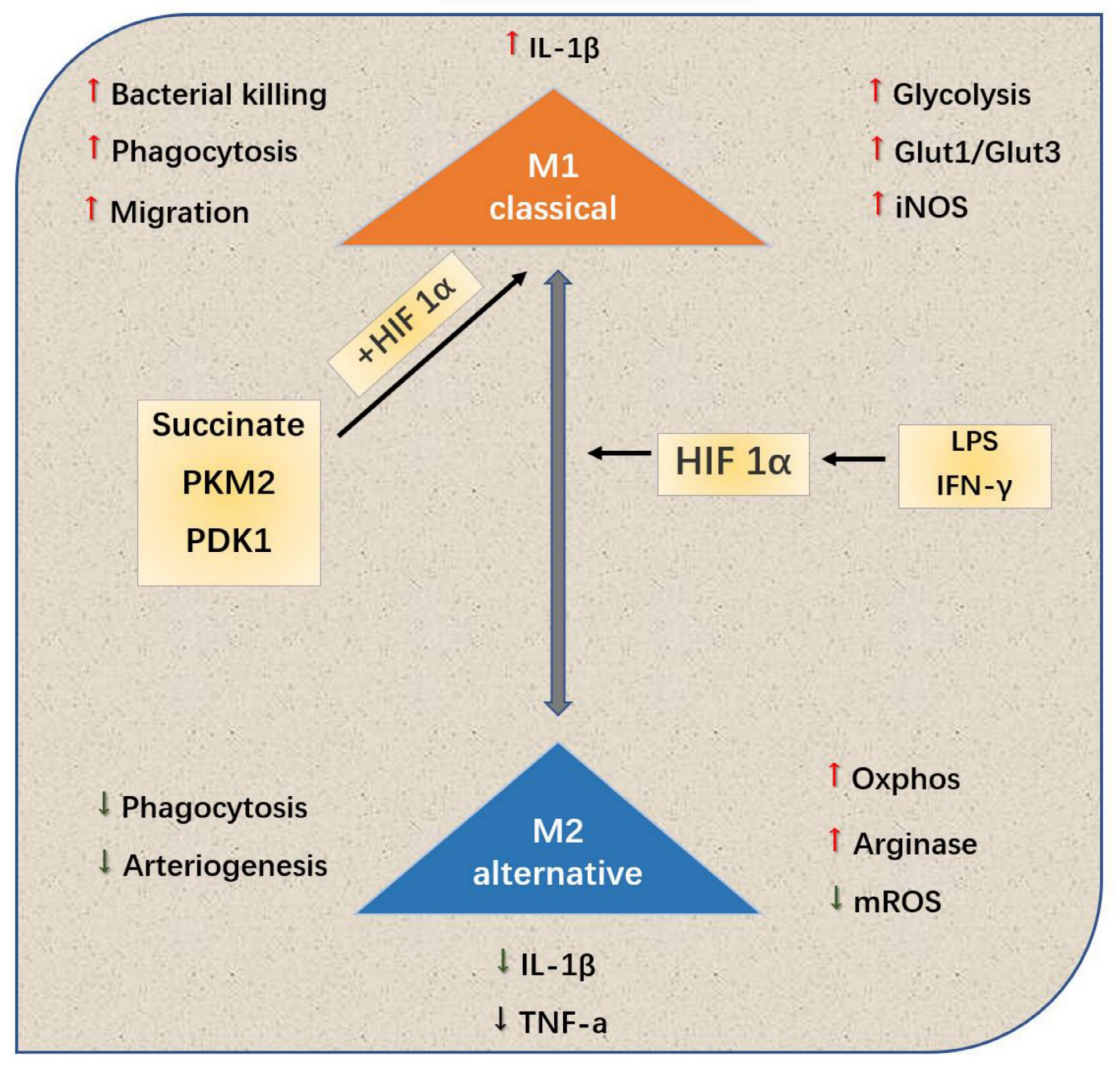
HIf-1a tends to M1 or typically activated cells, this polarization leads to changes in the production and metabolism of inflammatory cytokines. In addition, metabolites and metabolic enzymes, including succinate, PDK1 and PKM2, can also play a role in the polarization of HIf-1a-dependent M1.

Different microenvironments, such as hypoxia, inflammation and toxicant, can polarize macrophages into type M1 or M2, thus affecting the process of PAH^67^. Kojima et al.^68^ used targeted therapy technique to selectively knock out the gene HIF-1a of myeloid cells of mouse C57BL/6, extracted the peripheral blood macrophages of the gene-knockout and wild mice, and adopted cobalt chloride to induce the expression of HIF-1a. From the levels of mRNA and protein, it is confirmed that the HIF-1a expression of peripheral blood macrophages of the gene-knockout one is significantly lower than that of the wild one, and the protein expression is basically missing. Cramer et al.^[69]^revealed the relationship among the hypoxia signal pathway, metabolism and polarization state of macrophages. Various HIF subtypes may regulate the polarization state of macrophages (and vice versa), thus affecting the inflammatory results (Fig. 3).

Tannahill et al.^[70]^regarded succinate as HIF-1a-dependent inflammatory signal: the succinate produced by macrophages was necessary for the stability of HIF-1a to generate cytokine IL-1b. In recent years, Palson-McDermott et al.^[71]^found that as an additional HIF-dependent regulator, PKM2 was transferred to the nucleus in the form of non-enzymatic activity, and interacted with HIF-1a to up-regulate IL-1b. In addition, such as glycolytic regulator pyruvate dehydrogenase kinase 1(PDK1), it is also conducive to HIF-1a-induced glycolysis of macrophages^72^. To conclude, HIF-1a subtype related to macrophage metabolism significantly regulates macrophage.

### 3.5 HIF-1a and chronic inflammation

In chronic respiratory diseases, inflammatory cells and factors are important factors to induce airway remodeling. Under hypoxia, HIF-1α can promote inflammation with more inflammatory factors produced, such as interleukin (IL-1, IL-9, IL-13), tumor necrosis factor-alpha (TNF- α), which promotes excess secretion of mucus and induce airway remodeling in COPD. The addition of HIF-1α also helps release IL-1β, IL-8, monocyte chemoattractant protein-1 and other inflammatory factors. Knocking out HIF-1α or inhibiting phosphatidylinositol 3-kinase (PI3K)/protein serine-threonine kinase (Akt)/HIF-1α can reduce the production of inflammatory factors induced by hypoxia^73^. On the contrary, inflammatory cells and factors can also stimulate the expression of HIF-1. Neutrophils express HIF-1 to maintain the survival of neutrophils, and macrophages also express it. What is the difference is that subtypes of HIF-1 can induce different polarization states of macrophages^74^. Inflammatory factors, such as interferon (IFN-γ), mainly activate transcription of HIF-1α through nuclear factor kappa-light-chain-enhancer of activated B cells (NF-κB). After IL-1β activates NF-κB signal to induce the expression of HIF-1α, HIF-1α can combine with the promoter of gene Muc5ac, promote mucus production and participate in airway remodeling^75^. Research shows that HIF-1α under the conditions of hypoxia and inflammation can activate NF-κB, and promotes immune inflammatory reaction, leading to the occurrence and development of HPH. Therefore, HIF-1α significantly promote the inflammatory reaction of HPH^76^.

To sum up, hypoxic pathway is closely related to chronic inflammatory process. Hypoxia can activate inflammatory pathway to induce inflammation and airway remodeling, and inflammatory microenvironment can also up-regulate HIF-1a to exacerbate hypoxia. The specific mechanism remains to be explored.

### 3.6 HIF-1a and right ventricular remodeling

HPH is a complex cardiopulmonary disease, beyond instigating pulmonary vascular remodeling, it further precipitates right ventricular remodeling. Clinically, right ventricular (RV) function emerges as a pivotal determinant of patients' long-term survival, with chronic hypoxia-induced right ventricular remodeling (RVR) characteristically correlating with a deteriorated prognosis in HPH. The RVR process is generally bifurcated into 'compensatory' and 'decompensatory' phenotypes. The former is chiefly characterized by right ventricular hypertrophy (RVH) and is accompanied by minimal RV dilation and fibrosis, indicative of RV functional compensation. In contrast, the latter phenotype, 'decompensatory' RVR, is typified by cardiomyocyte apoptosis, fibrosis, progressive dilation, and a decrease in RV capillary density, collectively contributing to diminished exercise capacity and cardiac output^77^. HIF-1α exerts a pivotal regulatory influence on the pathogenic mechanisms underpinning the progression of pulmonary arterial hypertension and ventricular remodeling in congenital heart disease^78^, however, the mechanisms facilitating RVR occurrence within a chronic hypoxic environment remains unclear.

Recent clinical and experimental research has identified several key structural and molecular determinants associated with "compensatory" or "decompensatory" RVR. These factors encompass capillary rarefaction, metabolic transition from oxidative metabolism to glycolysis, excessive sympathetic nervous system activity, and upregulation of fibrotic pathways^79^.

Reduced microvascular density is considered a requisite factor in "decompensatory" RVR, and previous studies have observed a relative decrease in capillary density in RV samples from PH patients and animal models of PH. Holscher et al. demonstrates^[80]^that cardiac-specific HIF-1 overexpression exacerbates pressure overload-induced myocardial remodeling and heart failure. They found increased expression of HIF-1α in the hearts of late-stage heart failure patients, suggesting that prolonged chronic upregulation of HIF-1α has a deteriorating effect on the heart. However, Wei et al.'s study^[81]^indicate that HIF-1 gene knockout exacerbates pressure overload-induced myocardial fibrosis, cardiomyocyte hypertrophy, reduced myocardial capillary density, and cardiomyocyte apoptosis, promoting the occurrence of heart failure, suggesting a protective role of HIF-1 in the hearts of pressure-overloaded mice. Smith et al.^[82]^explored the impact of intracellular HIF-1α expression on HPH and observed that chronic hypoxia reduced stroke volume and cardiac output in wild-type mouse hearts, but not in HIF-1α-deficient mouse hearts. Simultaneously, the absence of HIF-1α in smooth muscle cells attenuated the rise in RV systolic pressure induced by chronic hypoxia, without improving right ventricular hypertrophy. Conversely, HIF-1α deficiency in cardiomyocytes exacerbated right ventricular remodeling, underscoring the protective role of HIF-1α in chronic hypoxia-induced RVR.

The results of the aforementioned studies indicate that HIF-1α plays a protective role in pressure overload-induced RVR. However, as pressure overload-induced RVR progresses to heart failure, increased HIF-1α expression exacerbates the condition. Therefore, there is a discrepancy in the role of HIF-1α in myocardial cells at different stages of HPH. Further elucidating the cellular and molecular mechanisms of HIF-1α in the pathogenesis of HPH will provide new evidence for the prevention and treatment of right ventricular remodeling and heart failure induced by pressure overload.

Additionally, research has suggested a potential linkage between mitochondrial metabolism, ischemia, and "decompensatory" RVR. Bekeredjian et al.'s study^83^, utilizing mice with cardiac-specific HIF-1α overexpression, provides evidence that elevated HIF-1α levels can result in reduced myocardial contractile function. During chronic hypoxia, the production of reactive oxygen species (ROS) can enhance the stability and increased expression of HIF-1α, further indicating that prolonged stable activation of HIF-1 has a worsening effect in chronic heart failure.

## 4. HIF-1a as a target for the treatment of HPH

In recent years, more and more drugs directly inhibiting the HIF-1a pathway have been found to improve HPH. However, these drugs have not been clinically approved yet^84^. HIF-1a regulates the occurrence of HPH under hypoxia. Since its regulatory mechanism was discovered, drug research and development for target HIF-1a has not stopped so far.

### 4.1 Treatment of HPH by using natural drugs to regulate HIF-1a

Due to their diverse components, natural drugs have multiple therapeutic effects on various diseases. According to the differences of active components and chemical structures, they are divided into flavonoids, saponins, anthraquinones, alkaloids, astragalus, saponins, etc. The content of HIF-1a in the body is also affected by regulating its synthesis, degradation, etc. (Table 1), thus effectively treating cancer, inflammation, etc.^85^

Resveratrol, a stilbene polyphenol with antioxidant, neuroprotective, cardioprotective, anti-inflammatory and anticancer effects, has been proven to inhibit HPH by down-regulating the expression of HIF-1 through MAPK/ERK1 and PI3K/AKT signaling pathways^86^. Tagitinin C inhibits the metabolic disorder caused by the increase of HIF-1 under chronic hypoxia and the Warburg effect of PASMC by reducing the content of the key enzyme PDK1 that increases the Warburg effect^87^. Gong Xiaonan's team has proved through experiments that Astragaloside IV may pass signal pathway of Calpain-1/HIF-1α to improve MCT-induced oxidative stress in PAH rats^88^.

Chrysin (CH) is a flavonoid compound with a large number of pharmacological activities. A study shows that CH significantly improves hemodynamic parameters of HPH rat model, such as right ventricular hypertrophy index. Its mechanism may be related to the down-regulation of HIF-1α, BMP4, TRPC1 and TRPC expressions in PASMCs, thus regulating Ca^2+^ to play a role of HPH protection^89^.

Tetramethylpyrazine (TMP), an effective component of traditional Chinese medicine Chuanxiong, has many biological activities, including improving microcirculation, protecting coronary artery, clearing free radicals, and anti-tumor^90^, ^91^. Li Youwei's team found^92^ that after TMP intervention, the level of rat HIF-1α, VEGF was significantly lower than that of the model group, indicating that TMP inhibited the increase of HIF-1α and VEGF levels induced by hypobaric hypoxia, thus reducing the proliferation of PASMC, and alleviating the occurrence and development of vascular remodeling and pulmonary hypertension.

Lycium barbarum polysaccharides (LBP) is an effective component extracted from Lycium barbarum^93^, which has the functions of controlling blood sugar, aging, oxidation and hypoxia resistances^94^. Zhu Y anni's team confirmed that levels of mRNA and protein of silent mating type information regulation 2 homolog 1 (SIRT1) of PASMCs treated with LBP under hypoxia decreased, and the levels of matrix metalloproteinase 9 (MMP-9), mRNA and protein of HIF-1α increased. It is suggested that LBP can stimulate the expression of gene SIRT1, resist the decrease of SIRT1 level after hypoxic treatment, and inhibit the increase of HIF-1α and MMP-9 under hypoxia, which may play a role in alleviating pulmonary vascular remodeling and pulmonary hypertension^95^.

Salidroside, a traditional Chinese medicine, is the active ingredient of rhodiola which has an anti-hypoxia effect and can be used for the treatment of high-altitude reaction^96^. Zhou Zhengguang's team confirmed that salidroside down-regulated the expression of HIF-1α in HPH rat model, indirectly inhibiting HIF-1α and up-regulating the role of VEGF of downstream target gene; it also reduced the damage of hypoxia to endothelial cells, improved the dynamic imbalance of pulmonary artery vasoconstriction and relaxation factors, and weakened the proliferation of pulmonary vascular smooth muscle; it also effectively inhibited the pulmonary vascular remodeling, and the development of HPH^97^.

Phytoestrogen, genistein (Gen) and daidzein (DD) are the main active components of soybean abnormal flavonoids, which have antioxidant and anti-inflammatory effects^98^, ^99^. Previous studies show that phytoestrogens can significantly lower the thickening of pulmonary vascular wall caused by hypoxia, and reduce HPH^100^. Yang Juan et al. found^[101]^that phytoestrogens might affect the expression of annexin A1 (AnxA1), a formyl peptide receptor agonist in hypoxic alveolar epithelial cells through pathways of HIF-1α and NF-κB; and phytoestrogens inhibited the expression of AnxA1 in hypoxic alveolar epithelial cells, thereby regulating the infiltration of inflammatory cells around the pulmonary vessels, and improving HPH.

Baicalin is a natural flavone with anti-thrombotic, anti-hyperlipidemic and anti-inflammatory effects. Huang S^102^ found that baicalin inhibited the expression of HIF-1α and aromatic hydrocarbon receptor (AhR), and reduced the proliferation and phenotype transformation of PASMC. Therefore, baicalin is expected to become a new drug for the treatment of PAH.

Echinaceoside (ECH) is a natural derivative compound with a structure of polyhydroxyphenol, which exists in many plants, such as cistanche deserticola, and has the functions of anti-oxidation, anti-inflammation, neuroprotection, liver protection and scavenging activity of nitric oxide free radical^103^. Gai XY et al.^104^ confirmed that ECH partially inhibited the proliferation of PASMC induced by hypoxia by reducing the expression of HIF-1a.

Caffeic acid phenylethyl ester (CAPE) is the main active ingredient in propolis, because it inhibits the activity of NF-κB; its anti-inflammatory effect has been widely known^105^. CAPE inhibits the hypoxia and the expression of PDGF-BB-induced HIF-1α by reducing the activation of AKT/ERK pathway, thus inhibiting the proliferation of PASMCs. In addition to NF-κB, HIF-1α is considered as an alternative target of CAPE. Thus, CAPE may be a promising therapeutic agent for PAH^106^.

Icariin (Ica) has pharmacological effects, including regulating immunity, anti-oxidation and anti-inflammation. It can inhibit the signal pathway of HIF-1α/TNF-a/NF-κB, alleviate pulmonary vascular remodeling and improve pulmonary artery and right ventricular hemodynamic abnormalities^107^.

Magnesium lhospermate B (MLB) is the main component of water extract of Salvia przewalskii Maxim, which has therapeutic effects on angina, cardiovascular injury, anti-inflammation, anti-oxidation, anti-apoptosis, etc. Wang et al.^108^ found that the expression of HIF-1α, proliferating cell nuclear antigen (PCNA), NF-κB, monocyte chemotactic protein-1 (MCP-1) and cyclin dependent kinase 4 (CDK4) of the HPH rat model after MLB treatment decreased, which ultimately inhibited the remodeling of pulmonary microvascular, and reduced mPAP and right ventricular hypertrophy index in HPH rat.

### 4.2 Chemotherapy scheme using HIF-1a as a target

At present, drug research and development using HIF-1a as a target are mainly aimed at the treatment of malignant tumors. However, inhibiting angiogenesis in the tumor microenvironment under hypoxia for HPH treatment has been drawn less attention.

Bortezomib can reduce the expression of HIF-1α under hypoxia. A further study shows that it can inhibit the proliferation of PASMC under hypoxia^109^. Etakalin hydrochloride can reduce the expression of HIF-1 in PASMC and pulmonary vessels under hypoxia, inhibit the proliferation of PASMC and promote apoptosis, thereby decreasing pulmonary artery pressure^110^. Braga CL et al. confirmed that the combined treatment of niclosamide and sildenafil/niclosamide reduced the expression of downstream target genes of signal transduction and transcription activating factor-3 (STAT3) in PEAC and PAAF. STAT3 is one of the main intracellular transcription factors involved in HPH vascular remodeling. This process is related to the fact that the combined treatment of niclosamide and sildenafil/niclosamide reduces the expression of STAT3 downstream targets of HIF-1, etc. in lung tissue^111^.

Celastramycin as a benzoyl pyrrole compound is originally found in bacterial extracts. It can reduce HIF-1α and NF-κB in PASMCs levels, thereby lowering the secretion of inflammatory factors and inhibiting the proliferation of PAH-PASMCs in a dose-dependent manner^112^.

Topotecan (TPT) can significantly inhibit hypoxic-induced PASMCs in normal rats. The up-regulation of HIF-1α and TRPC1/4/6 of rat'pulmonary artery in PAH model can effectively reduce the remodeling of its pulmonary artery in hypoxic-induced PAH model, improve its hemodynamics, and reduce right ventricular hypertrophy and ventricular wall thickening^113^.

The latest research has discovered that novel derivatives of bosentan, namely 17d, 16j, and 16h, endothelin receptor antagonists, can dose-dependently decrease HIF-1α levels in PAH rats^114^. A novel lysosomal autophagy inhibitor (ROC-325) can downregulate HIF-1αprotein expression in PASMCs, activate the eNOS-NO signaling pathway, and inhibit autophagy, thus suppressing pulmonary vascular remodeling, inducing pulmonary vasodilation, and alleviating the occurrence and progression of pulmonary arterial hypertension^115^.

## 5. Conclusion and prospects

HPH is resulted from the interaction of PASMCs, vascular endothelial cells, fibroblasts and macrophages under hypoxia, and the joint participation of various factors.HIF-1a as an important transcription and regulation factor that regulates angiogenesis, glucose metabolism and hematopoiesis, plays a key regulatory role in the occurrence of HPH.Although a large number of studies have proved that HIF-1a promotes the development of airway remodeling in chronic respiratory diseases, the relevant mechanism is not clear. Therefore, inhibiting pulmonary vascular remodeling by regulating HIF-1a is an effective target for the treatment of HPH.

It is shown that many natural drugs can affect the content of HIF-1a, and reduce the side effects of other drugs when used in combination with other marketed drugs as well. The pharmacological effects of these natural drugs are primarily achieved by inhibiting the proliferation of PASMCs, promoting PASMC apoptosis, regulating vasoconstrictive factors, suppressing oxidative stress, alleviating inflammatory responses, and modulating autophagy. These natural medicines mainly exert their therapeutic actions by curbing PASMC proliferation, facilitating PASMC apoptosis, adjusting vasoconstrictor agents, counteracting oxidative stress, mitigating inflammation, and regulating autophagy. At present, well-known monomer components affecting HIF-1a mainly include flavonoids, terpenes and glycosides. Therefore, still more active monomers exist in natural drugs and traditional Chinese medicine.

The evidence collected so far shows that chemical drugs and natural drugs can treat various diseases (especially tumors) by regulating HIF-1a^85^. However, most experimental results only come from animal or in vitro cell experiments. Limited and single HPH animal models may not be able to verify the efficacy of natural drugs. Nowadays, most studies only focus on the inhibitory effect of natural drugs compound on HIF-1a. However, few studies deeply explore their effects on the specific process of HIF-1a synthesis. In addition, how natural compounds regulate key genes, proteins, and even microRNA and lncRNA still need to be highly concerned. Extensive pharmacological experiments show that microRNAs also regulate proliferation, migration, and apoptosis-related signaling pathways by activating specific targets. Therefore, in the future research, it is suggested to pay more attention to the promotion of natural drugs on HIF-1a, and further explore its mechanism, pharmacokinetics and safety evaluation by combining in vivo and in vitro research methods. It is believed that with the in-depth study of HIF-1a regulation mechanism, this review can provide new ideas for HPH intervention.

## Figures and Tables

**Table 1:** Natural drugs that can inhibit HIF-1α in HPH.

Compounds	Original Plants	Chemical Structure	Experimental Models	Targets & Mechanisms	Reference
Resveratrol	Veratrum grandiflorum O, skins of grape (50- 100 μg/mL), peanuts, bilberries, blueberries and cranberries, et al	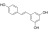	Male Sprague-Dawley rats, weighing 260 ± 9.6 g	Down-regulate the expression of HIF-1a through MAPK/ERK1 and PI3K/AKT signaling pathways	[86]
Tagitinin C	Tithonia diversifolia, Helianthus annuus	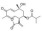	PASMC	Reduce the content of the key enzyme PDK1 that increases the Warburg effect and HIF-1	[87]
Astragaloside IV	Astragalus propinquus Schischkin	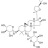	Male Sprague-Dawley rats, weighing200 ± 10g	Pass signal pathway of Calpain-1/HIF-1α to improve oxidative stress	[88]
Chrysin	citrus fruits, honey, propolis	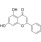	PASMC	Down-regulation of HIF-1α, BMP4, TRPC1 and TRPC expressions in PASMCs	[89]
Tetramethylpyrazine	Ligusticum chuanxiong Hort	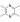	Male Sprague-Dawley rats, weighing 250 ± 20g	Inhibit the increase of HIF-1α and VEGF levels	[91]
Lycium Burbarum polysaccharides	Lycium barbarum L	None	PASMC	Resist the decrease of SIRT1 level after hypoxic treatment, inhibit the increase of HIF -1α and MMP-9 under hypoxia	[93]
Salidroside	Rhodiola rosea L.	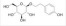	Male/Femal Wistar rats, weighing 150±10g	Indirectly inhibit HIF-1α and up- regulate the role of VEGF of downstream target gene	[96]
Genistein, Daidzein	soybean	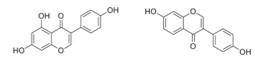	Human alveolar epithelial cells	Affect the expression of annexin A1, a formyl peptide receptor agonist in hypoxic alveolar epithelial cells through pathways of HIF -1α and NF- κB	[95]
Baicalin	Scutellaria radix (Huang Qin)	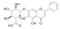	PASMC	Inhibit the expression of HIF- 1α and aromatic hydrocarbon receptor, reduce the proliferation and phenotype transformation of PASMC	[96]
Echinacoside	Cistanche tubulosa	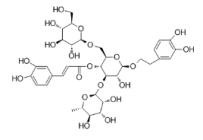	PASMC	Inhibit the proliferation of PASMC induced in hypoxia by reducing the expression of HIF -1a	[103]
Caffeic acid phenethyl ester promotes	propolis	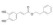	PASMC	Inhibit the hypoxia and the expression of PDGF -BB- induced HIF -1α by reducing the activation of AKT/ERK pathway	[105]
Icariin	Epimedii	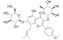	Male C57 mice, weighing 20-25g	Inhibit the signal pathway of HIF-1α/TNF -α/NF-κB	[107]
Magnesium lithospermate B	Salviae miltiorrhizae	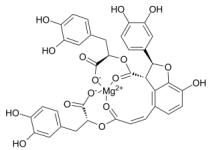	Male Sprague- Dawley (SD) rats, weighing 120-160 g	Down-regulate the expression of HIF- 1, NF-κB, monocyte chemotactic protein-1, (CDK4) of the HPH rat model	[108]

## Abbreviations

HIF: hypoxia inducible factor
PAH: pulmonary hypertension
HPH: hypoxia induced pulmonary hypertension
HPV: hypoxic pulmonary vasoconstriction
HPVR: hypoxic pulmonary vascular remodeling
HRE: hypoxia response element
PASMCs: pulmonary artery smooth muscle cells
EPO: erythropoietin
ARNT: aryl hydrocarbon receptor nuclear translacator
PHD: prolyl hydroxylase-domain protein
ROS: reactive oxygen species
VEGF: vascular endothelial growth factor
VSMC: vascular smooth muscle cell
EndMT: endothelial-to-mesenchymal transition
TGF-β: transforming growth factor beta
ET: endothelin
iNOS: inducible nitric oxide synthase
PAEC: pulmonary artery endothelial cell
PCNA: Proliferating Cell Nuclear Antigen
ncRNA: non-coding RNA
RUNX2: Runt-related Transcription Factor 2
KLF5: Krüppel-like factor 5
SENP-1: SUMO-specific protease-1
ADM: adrenomedullin
FDG: Fludeoxyglucose
GLUT1: Glucose transporter 1
PKM2: Pyruvate kinase isozyme type M2
G6PD: Glucose6 Phosphate Dehydrogenase
PDGF: platelet-derived growth factor
Drp1: dynamin-related protein 1
AKG: Alpha-ketoglutarate
PFKFB3: 6-phosphofructokinase-2isoenzyme 3
PI3K: Phosphatidylinositol-4:5-bisphosphate 3-kinase
Akt: protein kinase B
E2: 17β-Estradiol
SMC: smooth muscle cell
α-SMA: α-smooth muscle actin
ESM-1: endothelial-cell-specific molecule-1
IL: interleukin
PAAF: pulmonary artery adventitia fibroblast
ECM: extracellular matrix
PDK: pyruvate dehydrogenase kinase
LPS: Lipopolysaccharide
PDK1: pyruvate dehydrogenase kinase 1
TNF-α: tumor necrosis factor-alpha
IFN-γ: interferon
NF-κB: nuclear factor kappa-light-chain-enhancer of activated B cells
CH: Chrysin
BMP-4: bone morphogenetic protein-4
TRPC: transient receptor potential cation channel
TMP: tetramethylpyrazine
LBP: Lycium Burbarum polysaccharides
SIRT1: silent mating type information regulation 2 homolog 1
MMP-9: matrix metalloproteinase 9
mPAP: mean pulmonary arterial pressure
DD: daidzein
AnxA1: Annexin A1
ECH: Echinacoside
CAPE: Caffeic Acid Phenethyl Ester
Ica: icariin
TLR4: Toll-like receptor 4
STAT3: signal transducer and activator of transcription 3
TPT: Topotecan
